# The role of co-design and co-creation methodologies in research supporting older adults living with HIV: A scoping review

**DOI:** 10.1371/journal.pone.0358263

**Published:** 2026-09-18

**Authors:** Esther Su, Luxey Sirisegaram, Hardeep Singh, Andrew D. Eaton, Sarah E. P. Munce, Paige Brown, Carolina Navas, Stuart McKinlay, Alice Zhabokritsky, Kristina M. Kokorelias

**Affiliations:** 1 Division of Geriatric Medicine, Department of Medicine, Sinai Health System and University Health Network, Toronto, ON, Canada; 2 Department of Occupational Science & Occupational Therapy, Temerty Faculty of Medicine, University of Toronto, Toronto, ON, Canada; 3 KITE, Toronto Rehabilitation Institute, University Health Network, Toronto, ON, Canada; 4 Temerty Faculty of Medicine, Rehabilitation Sciences Institute, University of Toronto, Toronto, ON, Canada; 5 Faculty of Social Work – Saskatoon Campus, University of Regina, Regina, SK, Canada; 6 Factor-Inwentash Faculty of Social Work, University of Toronto, Toronto, ON, Canada; 7 Institute of Health Policy, Management and Evaluation, University of Toronto, Toronto, ON, Canada; 8 Undergraduate Medical Education, Temerty Faculty of Medicine, University of Toronto, Toronto, ON, Canada; 9 Infectious Diseases, Department of Medicine, University Health Network, Toronto, ON, Canada; 10 Division of Infectious Diseases, Department of Medicine, University of Toronto, Toronto, ON, Canada; 11 CIHR Canadian HIV Trials Network, Vancouver, BC, Canada; University of Foggia: Universita degli Studi di Foggia, ITALY

## Abstract

**Introduction:**

This study aims to synthesize existing literature on co-design and co-creation methodologies that support older adults living with HIV and to identify opportunities and challenges for applying these approaches in geriatric HIV contexts.

**Methods:**

A protocol for this scoping review was published elsewhere; we also describe our methods below. The Joanna Briggs Institute (JBI) Manual for scoping reviews guided our approach, including database searching, title/abstract and full-text screening, data extraction, and analysis. We followed PRISMA recommendations for evidence screening and selection.

**Results:**

Our search identified 2,734 records from databases and no articles from grey literature. Across included studies, we identified themes describing how co-design and co-creation are used with older adults living with HIV, along with persistent challenges in implementation. Co-designed digital interventions may be less accessible for older adults who have lower digital literacy or limited access to devices or internet, and participants often represent a subset of older adults who are more able to engage in workshops or interviews or who have lower health status. Advisory committee members emphasized the importance of diverse participation and attention to accessibility throughout the process.

**Conclusions:**

This review synthesizes evidence on co-design and co-creation for older adults living with HIV and highlights implications for practice and future research participation and engagement in this population. Findings point to the need for clear processes, inclusive recruitment, and attention to digital access and support when implementing co-designed interventions in geriatric HIV care.

## Introduction

Older adults living with HIV represent a growing and unique population in the global HIV epidemic [1–3]. With advancements in antiretroviral therapy (ART), individuals diagnosed with HIV now have the potential to live longer, but this longevity brings unique challenges from the convergence of ageing and HIV [4,5]. For example, in addition to HIV management, older adults living with HIV often must also navigate the health challenges of chronic comorbidities, polypharmacy including ART drug interactions, and geriatric syndromes affecting up to 30–50% of HIV patients aged over 50 [4,6].

Potentially further complicating the ageing process for this patient population is the two-fold stigma from both ageing and HIV status, leading to increased social isolation and decreased engagement in appropriate health care [7–11]. Stigma experienced by older adults living with HIV presents significant barriers to engaging in care and can moreover extend to limiting participation in research studies. For example, there may be fear that research involvement could expose them to discrimination, jeopardize their privacy, or reinforce negative societal attitudes about their health status [11,12]. This is particularly true for individuals who have lived with HIV for decades, many of whom have endured decades of social marginalization and the trauma of living through earlier periods of the epidemic, when AIDS-related mortality was higher. The continued stigma of HIV being linked to specific social groups (e.g., men who have sex with men, intravenous drug users) exacerbates the experiences of discrimination [11]. For this population, the act of engaging in research may trigger feelings of vulnerability and further stigmatization, limiting their willingness to contribute their perspectives or experiences [12]. The combination of these factors often results in an underrepresentation of older adults with HIV in research, contributing to a lack of healthcare interventions tailored to meet the needs of this population [13]. Therefore, developing health care services tailored to meeting the unique needs of older adults living with HIV is essential, warranting research which supports the increased involvement of this growing patient population [14].

Co-design and co-creation are participatory methodologies that offer a unique and powerful way of addressing these barriers, fostering a collaborative, inclusive, and supportive environment for older adults living with HIV. These methodologies emphasize the inclusion and partnership of end-users and/or other stakeholders [15]—older adults living with HIV, their caregivers, and healthcare providers—in the design and implementation of services and interventions [16]. Co-design typically involves cycles of idea generation, prototyping, testing, and refinement, with direct partnership from users at each stage of designing a solution to a specific problem [15]. Co-creation, while similar to co-design, emphasizes not only the design process but also the joint creation of value between knowledge users [15]. Co-creation is more encompassing, referring to the active participation of users and service providers, from defining the problem to the continuous improvement of services and interventions, focusing on fostering collaboration and engagement throughout the entire service process [15]. In health care, co-creation ensures that services are responsive to the evolving needs of patients, including those living with chronic conditions such as HIV [16]. These methodologies align well with the principles of person- and family-centered care (PFCC) for ensuring that care is responsive, respectful, and tailored to individual preferences [17,18]. Likewise, co-design approaches, when applied to the context of older adults living with HIV, have the potential to generate solutions that address the specific challenges faced by this population, such as stigma, social isolation, and the need for integrated care models [16]. By involving individuals with lived experience in the development of healthcare interventions, co-design helps to ensure that the services or solutions created not only address their needs but also empower them to take an active role in shaping their own care.

Beneath the potential benefits of co-design and co-creation for older adults living with HIV lie challenges in implementing these methodologies. Older adults living with HIV have diverse health, social, and cultural needs, which can make it difficult to design universal solutions [14]. However, involving a broad range of older adults living with HIV in the co-design process can help ensure that interventions are more inclusive and adaptable [14]. Furthermore, co-design has previously been successfully applied to older adult populations in the context of developing interventions for active ageing and ageing in place; however, previous systematic reviews have found high heterogeneity in the implementation of co-design in these studies, finding that there was a notable absence of systematic approaches for participatory methodologies involving older adults [19–21].

Currently, studies on the application of co-design and co-creation methodologies specifically for older adults living with HIV are limited, and the existing literature often fails to synthesize findings in a way that can provide clear guidance for practitioners and researchers [16]. Thus, there is a lack of comprehensive frameworks and strategies to guide their implementation in this specific context [16]. These gaps present an opportunity to explore and consolidate the evidence on co-design and co-creation in HIV care, offering a foundation for effective service delivery and improved health outcomes for older adults living with HIV [16]. This scoping review aims to synthesize the literature on co-design and co-creation research involving older adults living with HIV, seeking to understand strengths, limitations, and considerations for future research. As research on co-design and co-creation involving older adults living with HIV remains limited and conceptually diverse, a scoping review is warranted to map the existing evidence, clarify key concepts, and identify knowledge gaps. Unlike systematic reviews, which evaluate effectiveness, a scoping review is suited to examining how and where these participatory methods have been applied in this emerging area of research. Evidence synthesis will be crucial for creating a cohesive body of knowledge that can inform the development of best practices and evidence-based guidelines for health services to meet the needs of this patient population [16].

## Materials and methods

A protocol for this scoping review was published elsewhere; [16] however, we also describe our methods below.

The Joanna Briggs Institute (JBI) Manual for scoping reviews guided the review process, including the Population, Concept, Context (PCC) framework for conducting scoping review studies [22,23]. The Sex- and Gender-Based Analysis Plus (SGBA+) framework was used to conceptualize different stages of the review [24]. The review followed a systematic process, including developing a search strategy, evidence screening and selection, data extraction, and analysis. The reporting of this review adheres to the PRISMA extension for scoping reviews (PRISMA-ScR) [25], with the completed PRISMA-ScR checklist provided in S1 Checklist.

### Stage 1: Developing a search strategy

The search strategy (see S1 Appendix) was developed using OVID Medline by the research team and an Information Specialist and Health Science librarian (CDC). The preliminary plan was peer-reviewed by another librarian using the Peer Review of Electronic Search Strategies (PRESS) Statement [26] and resulted in no changes to the strategy. Searches were conducted on May 17, 2024 and updated on June 10, 2025 across multiple databases: NLM’s PubMed OVID Embase  +  Embase Classic, EBSCO’s CINAHL Complete, Clarivate’s Web of Science Core Collection, and Elsevier’s Scopus by the Information Specialist and Health Science librarian. Relevant grey literature was identified through handsearching using search terms similar to those used in the scientific search. This was done via Google Scholar, Open Grey, open Google searches, and relevant websites, including the WHO, UK National Research Register, CADTH’s “Grey Matters,” the New York Academy of Medicine’s Grey Literature Report, the Canadian Medical Association InfoBase, and the National Institute for Health and Care Excellence–Guidance. The search was inputted into an Endnote library for reference management, and duplicate articles were removed [27]. We searched the reference lists of included articles and relevant reviews (i.e., reviews not included in this study but were flagged during screening) [28]. All database strings and dates are provided in S1 Appendix.

### Stage 2: Evidence screening and selection

Covidence software was used to manage evidence screening and selection, allowing for a double-blind review process [29] with inclusion criteria based on the PCC framework [30] (see Table 1 in Brown et al.[16]). The inclusion criteria required studies to focus on individuals aged 50 or older living with HIV who participated in co-design or co-creation methods. Studies were eligible if they described co-design or co-creation research and employed empirical data collection (qualitative, quantitative, multi-methods or mixed methods, excluding case reports). Studies published in full-text English within the past 20 years from any healthcare setting or country were considered. Studies on conceptual models, unimplemented research, or non-empirical literature (e.g., conference abstracts and theses) were excluded. Two reviewers independently assessed articles based on eligibility criteria during level 1 (title and abstract) and level 2 (full-text) screening stages. The senior author (KMK) resolved any conflicts.

**Table 1:** Characteristics of Included Studies.

Study (year, country)	Study objective	Engagement level	Older adults (n, ≥ 50 years)	Total sample (N)	Methods	Output
Clarke et al., 2023 [31], Tanzania	To identify participant priorities for future HIV ageing research conducted in Tanzania	Consultation only	30	30	Single workshop in Swahili led by local facilitators	Ranked research priorities list
Kanazawa 2021 et al.[32], United States	To obtain participant perspectives on conducting HIV cure research in end-of-life patients	Consultation only	≥10*	16	20 interviews + 3 focus groups (virtual)	Ethical and practical considerations for end of life HIV cure research
Nguyen et al., 2017 [33], United States	To obtain participant perspectives for planning a prospective cohort study in Coachella Valley on HIV ageing	Consultation only	18	18	Three focus groups at an HIV services centre	Design inputs for a prospective cohort study
Kokorelias et al., 2025 [34], Canada	To co-design a HIV virtual care model for older adults living with HIV	Consultation only	19	19	Three-phase co-design: interviews, focus groups, and workshops (12 months)	Virtual care model components and implementation insights
Marent et al., 2018 [35], European Union	To co-design an mHealth platform for HIV care, supporting follow-up and self-management in five European clinics	Consultation only	28	97	Ten workshops + 20 interviews across five countries	Platform feature set and implications for use
Njie-Carr et al., 2018 [36], United States	To obtain participant input for developing an mHealth app to support older women living with HIV	Partnership (CAB) + participants (focus groups)	10 (participants)	10 (participants) **	Two focus groups; CAB meeting (~2 h)	Prioritized app features and content
Solomon et al., 2016 [37], Canada	To develop rehabilitation recommendations for older adults living with HIV	Partnership (research team) + consultative external endorsement	11 (3 partners; 9 external people living with HIV)	11 (older adults living with HIV)	Research-team meetings (evidence review, GRADE); external endorsement survey	Rehabilitation recommendations

Notes: *Age bands reported imply at least 10 of 16 participants were ≥50; 25% age unspecified. **Row reports participant (focus-group) N; CAB members are partners. Abbreviations: CAB = community advisory board; GRADE = Grading of Recommendations, Assessment, Development and Evaluations

### Stage 3: Data extraction

Data extraction was facilitated using a customizable form in Covidence [38]. Initially, two reviewers independently extracted data from a random sample of five included studies and then independently extracted data. The senior author (KMK) reviewed all the extracted data for accuracy. Data extraction involved extracting information on study characteristics, including author, year, study type, setting, country, methods, methodology, characteristics of intervention, delivery method (e.g., virtual, in-person, or telephone), participant characteristics, co-design partner characteristics, provider characteristics, results, and key conclusions used in the study. Participant and partner details were categorized according to the SGBA+ framework, including considerations of sex, gender, and other identity constructs [24]. Appraisal for risk of bias and study quality was not performed, consistent with the Joanna Briggs Institute Manual [22].

### Stage 4: Data analysis

Data analysis included quantitative and qualitative methods, summarizing results numerically and through content analysis [39]. Line-by-line coding was conducted to develop descriptive categories reflecting the content of included articles [40]. Quantitative analysis involved descriptive statistics to summarize study characteristics (e.g., year of publication, study design, geographic setting, population characteristics, and types of co-design or co-creation methods used). These data were tabulated to illustrate trends, patterns, and distribution across the literature base. Qualitative analysis was conducted through an inductive content analysis approach to identify, categorize, and synthesize key themes related to the implementation, facilitators, barriers, and outcomes of co-design and co-creation involving older adults living with HIV. Line-by-line coding of the full texts was undertaken to capture relevant concepts, with descriptive categories developed to reflect the range and depth of the content within included studies. The first and last author led content analysis using NVivo software [41], with coding verified independently by a second reviewer. Coding consistency and reliability were ensured through independent verification by a second reviewer, followed by iterative discussions among the research team to resolve discrepancies and refine the coding framework. Through this collaborative process, the team developed higher-order themes that synthesized the state of knowledge and identified conceptual and empirical gaps in the literature. This was followed by a discussion among the research team to address similarities and differences in the coded data.

### Stage 5: Consultation and Co-Creation

We embraced a co-creation approach, as outlined by Pollock et al. (2022), to gather feedback on our search strategy, maintaining this collaborative effort throughout the review process [42]. The co-creation panel—comprised of a 10-person advisory committee including experts in HIV care, service providers, and knowledge users—met on multiple occasions through structured discussions, scheduled review sessions, and email correspondence. The committee also included three women in their 70s living with HIV who brought critical lived-experience perspectives, including those with severe mobility challenges, blindness, and hearing impairment.

Review sessions occurred approximately every two months over the course of the review and were used to discuss key methodological steps, including refinement of the search strategy, inclusion criteria, and data extraction framework. These sessions emphasized co-learning and shared decision-making, ensuring that the evolving analytical and interpretive processes reflected the perspectives of both academic and community members.

In the final stages of manuscript preparation, we conducted a comprehensive virtual consultation and co-creation exercise to gather additional feedback and insights from our advisory committee. During the 2-hour virtual consultation, we presented a manuscript draft. We solicited their input on various aspects of the findings, including the interpretation of results, clarity of conclusions, and implications for practice and policy. Their constructive feedback was invaluable in refining the manuscript and ensuring its coherence and relevance. Specifically, their input helped to clarify specific findings, identify nuanced interpretations, and strengthen the overall argumentation. The discussion section details how our panel provided feedback and interpreted the results. All individuals were compensated for their time.

## Results

### Overview

**Fig 1:**
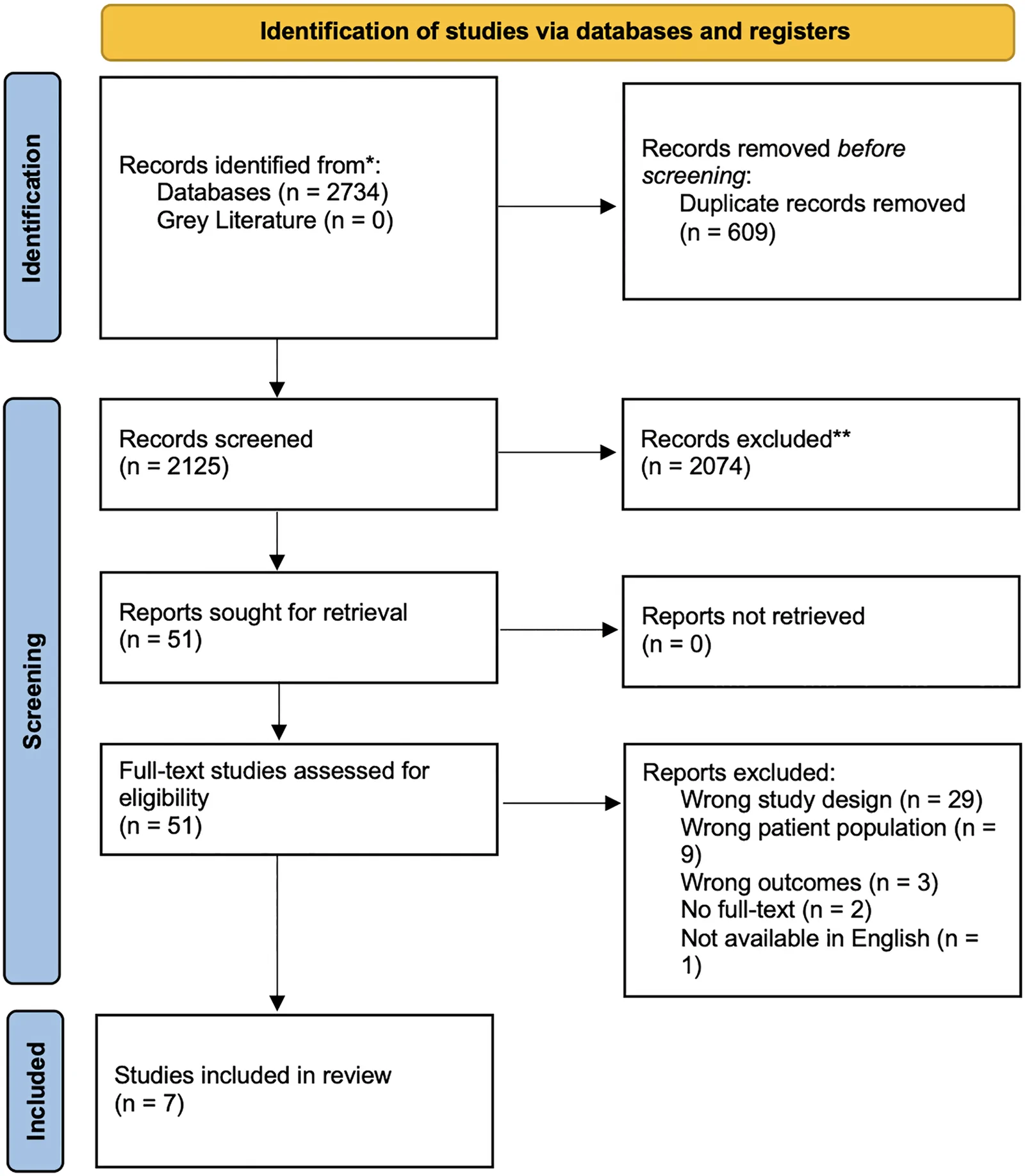
PRISMA flow diagram.

Our search identified 2734 articles from databases and no articles from grey literature. After removing duplicates, 2125 titles and abstracts were screened against inclusion and exclusion criteria, resulting in 51 studies eligible for full-text review. Following retrieval and assessment of full-text articles, we included a total of 7 studies (see Fig 1). The small number of included studies likely reflects both the emerging nature of research applying co-design and co-creation methodologies to older adults living with HIV and the stringent eligibility criteria employed in this review. Specifically, studies were required to explicitly engage older adults (aged 50+) living with HIV as active partners in the co-design or co-creation process rather than as passive participants or end-users, which excluded many descriptive or non-participatory studies. Study-level characteristics are summarized in Table 1. While this approach ensured conceptual precision and relevance to our objectives, it may have limited the number of eligible studies. This constraint is acknowledged as a limitation of the review but also highlights a significant gap in the literature and the need for more participatory research involving this population.

Collectively, the seven studies included 127 older adults living with HIV, ranging from 10 to 30 participants across studies. Three studies were conducted in the United States [32,33,36], one in Tanzania [31], two in Canada [34,37], and one across the European Union [35]. Published between 2016 and 2025, the seven studies engaged older adults living with HIV using a range of participatory research methodologies including co-design, community engagement and involvement (CEI), patient and public involvement (PPI), community-based participatory research (CBPR), experience-based co-design (EBCD), and community advisory boards (CAB). However, the level of engagement varied widely. Only two studies [36,37] described using a true co-design approach where older adults were integrated into advisory committees or project teams. The remaining five studies [31–35] engaged older adults as study participants (e.g., workshops, interviews, focus groups).

The stated objectives were generally related to either research planning or health intervention design. Two studies sought participant input on how future HIV ageing research should best be conducted, citing the novelty of the research areas as rationale for using a participatory approach [31,32]. Another paper aimed to engage community members in a co-design a prospective cohort study on ageing with HIV in California, however, participants were consulted rather than involved as partners [33]. Two studies were conducted to develop mobile app health interventions: one convened a CAB alongside focus groups to inform the design of an mHealth app for older women living with HIV [36], representing one of the two examples of partnership-based co-design, while another engaged participants in workshops and interviews to co-design an mHealth platform for supporting follow-up and self-management in HIV care, without any specific decision-making roles for older adults [35]. Also exploring digital interventions, a study that sought to co-create culturally appropriate virtual care models for older adults living with HIV [34]. Finally, a Canadian study used a partnership approach by integrating older adults as members of the research team to formulate rehabilitation-related recommendations in addition to other engagements with study participants [37].

### Co-creation methods

Workshops were conducted in three studies [31,34,35], individual interviews in three [32,34,35], and focus groups in four [32–34,36]. In one study, workshops were intentionally selected for feasibility and were conducted according to the James Lind Alliance framework, emphasizing inclusivity, transparency, and a commitment to using and contributing to the evidence base [31]. A 3-phase co-design process in another study followed EBCD methodology, with workshops being guided by a modified 4D Model of Appreciative Inquiry to focus on strengths, ideals, co-creation, and implementation [34]. Where a mix of interviews and workshops/focus groups was used, the same structured interview guide was used for consistency and ease of consolidating study findings [32].

The size of workshops and focus groups ranged from 5 to 30 participants and were conducted in person at accessible locations (e.g., schools, churches, service centres, community partner offices, hotels, clinics) [31–36] or virtually on a conferencing platform [32]. These activities were structured with interview guides and led by facilitators, overseen by healthcare professionals (e.g., clinicians and nurses), members of the research team, community partners, or facilitators with local cultural and language experience [31–35], and mainly centered around group discussion. In one study, participants engaged in group discussions to produce a list of HIV ageing research priorities, after which the list items were ranked by voting in the form of hand-raising [31]. In some studies, open-ended questions were used to stimulate discussion [32,35] while others involved visual tools (e.g., concept mapping, word clouds) [34].

Group-based co-creation activities did not exceed 2.5 hours, and individual interviews were shorter, lasting for an average of 46 minutes in one study [35]. One study conducted 3 phases of interviews and workshops over the span of one year, with the inclusion of both new and returning participants [34]. With the exception of Kokorelias et al.[34], workshops or focus groups were conducted as one-time events.

### Co-design methods

Co-design methods included the formation of project teams and advisory boards incorporating older adults living with HIV [36,37]. In one study, older adults living with HIV acted internally as members of the research team and, in subsequent stages, externally in consultation for finalizing the recommendations [37]. Researchers noted that community involvement in research processes can foster empowerment for partners, promotes relevance, and facilitates partnerships [37]. In another study, a CAB brought together researchers, clinicians, older adults living with HIV, and technology experts to ensure that the co-designed mobile health intervention would be sustainable and relevant for the target population [36]. Partners were not involved in data analysis or knowledge dissemination, but instead only advised on app features and design components [36].

### Recruitment and characteristics of co-design partners

Overall, demographic characteristics [43] of partners were rarely reported in included studies and instead (see Table 2), detailed characteristics were provided primarily for participants (see Table 3).

**Table 2:** Partners/governance summary (partnership-based studies only).

Study (year, country)	Governance structure	Composition (roles; # people living with HIV)	Demographics reported?	Decision role(s)	Supports/ compensation	Notes
Njie-Carr et al., 2018 [36] (US)	CAB	Researchers, clinicians, tech experts, city health; people living with HIV included	No (roles only)	Advice on content/features	Honoraria reported ($50 in-person; $30 phone)	CAB meeting ~2h; phone participation allowed
Solomon et al., 2016 [37] (Canada)	Research team + external endorsement	Team: 3 people living with HIV + clinicians, researchers, policy; external: people living with HIV + health professionals	No (counts/roles)	Evidence review, appraisal, drafting; endorsement	Not reported	Authors recommend clear ToR/decision rules

Abbreviations: CAB = community advisory board; ToR = terms of reference.

**Table 3:** Participant characteristics – condensed PROGRESS-Plus (Place, Race/ethnicity, Occupation, Gender/sex, Religion, Education, Socioeconomic status, Social capital).

Study (year, country)	Setting (place/region)	Age	Sex and Gender	Race/Language	Education/SES	Other context
Clarke et al., 2023 [31], Tanzania	Urban; single region	51-81 (median 68)	60% women	Activities in Swahili; race NR	NR	NR
Kanazawa 2021 et al.[32], United States	Multi-source recruitment	47-78 (median 58)	68.8% male	62.5% White; 31.3% Black; small % Hispanic or American Indian	NR	NR
Nguyen et al., 2017 [33], United States	Single region (Coachella Valley)	56-69 (mean 62)	89% male	NR	NR	Mean 26 years living with HIV
Kokorelias et al., 2025 [34], Canada	Urban; single region (Ontario)	50-52% ≥ 65 (across phases)	58–71% male; 25–30% female; small % other genders	43–60% not English first language; diverse ethnicities	<HS 20–25%; college/uni 30–42%; grad/prof 20–25%; 64–80% < $30k CAD	Multi-phase (1 year)
Marent et al., 2018 [35], European Union	Five countries; clinics, hotels, offices	Overall mean 43.7 (28.9% ≥ 50)	80.4% male	Multiple cultural+ linguistic backgrounds	NR	NR
Njie-Carr et al., 2018 [36], United States	Urban; community sites	58-69 (mean 62.8)	100% female	Black	All ≥ HS (mean 14.7 years); median $287.30/week	Comorbidities reported
Solomon et al., 2016 [37], Canada	Multi-stakeholder networks	NR	NR	NR	NR	Endorsement survey

Abbreviations: NR = not reported; < HS = less than high school; uni = university; grad/prof = graduate/professional degree.

**Recruitment and setting.** Study participants were recruited from HIV clinics, hospitals, and community agencies**.** In one study from the United States [36], older women living with HIV participated in focus groups, and two participants were involved in the CAB, alongside academic researchers (n = 2), clinicians (n = 3), technology experts (n = 2), and a city health department worker (n = 1). In the other Canadian study [37], three older adults living with HIV acted as members of the research team while others were involved in an external endorsement survey.

**Race and ethnicity.** In the U.S.-based study, all focus group participants self-identified as Black women aged 50 years or older [36]. Race or ethnicity of CAB members and partners were not reported in either study [36,37].

**Gender, sex, and age.** Only one study recruited older adults exclusively [36]. Focus group participants in one study all identified as women aged 58–69, with a mean age of 62.8 years [36]. Information about partner gender, sex, or age was not reported in either study [36,37].

**Education and socioeconomic status (SES).** In Njie-Carr et al., (2018), all participants completed high school, with 14.7 years as the mean number of years in school. Education and SES of partners were not reported [36,37].

### Recruitment and characteristics of co-creation participants

**Recruitment and setting.** Participants were often recruited from HIV clinics and hospitals, sometimes supplemented by academic institutions, community advisory networks, and local community agencies [31,32,34–36]. Within this subset (n = 5), four studies were conducted in urban settings in high-income settings [32–35], while the remaining study was conducted in a single urban site in Tanzania [31]. In three studies, recruitment was limited to a single geographic region [31,33,34]. Overall, recruitment favoured individuals already engaged in HIV care or community networks.

**Race, ethnicity, culture, and language.** Race or ethnicity information of participants was reported by three studies; however, the data were not stratified by age [32,34,35]. One study conducted in the United States reported that 10/16 participants (62.5%) were Caucasian and 5/16 (31.3%) were Black [32]. A study in Canada that purposively sampled participants included participants from a variety of ethnic and linguistic backgrounds, with 43–60% of participants not speaking English as a first language [34]. One study conducted across five clinics in Europe noted that participants were from multiple cultural and linguistic backgrounds [35].

**Gender, sex, and age**. Four studies from this subset recruited exclusively older adults living with HIV [31,33,34,36], while the remaining studies recruited people living with HIV without age requirements [32,35]. Participant ages typically ranged from late 50s to early 60s. In one study conducted in Europe, 28.9% of participants were over 50 years old [35]. In four of the seven included studies, male participants were over-represented (see Table 3), ranging from 58% to 89% of the overall participant sample [32–35]. One notable exception was a Tanzania-based study that included 60% women-identifying participants, which the authors describe as being due in part to the higher prevalence of HIV in women compared to men in this country [31]. Purposive sampling was used in three studies to specifically seek out female participants [35], individuals with prior knowledge of the research topic [32], or diversity across a combination of demographic dimensions [34].

**Education and SES.** Little information was reported on participants’ occupation or education levels, although one study described recruiting participants from a mixture of demographics including sex and educational/occupational backgrounds [31]. Demographic data collected in one Canadian study showed, with some variation between study phases, that 20–25% of participants had an education level lower than high school, while 30–42% of participants had at least a degree or diploma from college or university, and 20–25% of participants had a graduate or professional degree [34].

### Challenges and limitations noted in studies using co-design and co-creation

**Study and process-related constraints.** Researchers reported several challenges in conducting co-design and co-creation studies involving older adults living with HIV. Pragmatic factors such as time and funding constraints limited both study participant recruitment and choices on study design [31,32,35], however, one study indicated that participatory methodologies were still feasible, observing high engagement and open discussion among workshop participants [31]. Additional process factors included pandemic-related disruptions that may have created imbalances in those who were able to participate [32], and the exclusion of healthcare providers and community agencies in co-design activities, potentially limiting the clinical and service delivery aspects in the finalized intervention [34].

**Participant-related challenges.** Four studies noted that study participants tended to have pre-existing knowledge of and experience with HIV research due to recruitment strategies relying on HIV clinics, community organizations, and connections with researchers [31,32,35,36]. Two studies raised concerns over the exclusion of patients who were more vulnerable, less healthy, unable to regularly engage in HIV services, or lacking access to transport [31,36]. Similarly, two studies reported that most co-design participants were already experienced users of digital technology, which may result in overestimating the utility of the co-designed digital interventions among less experienced users [34,35].

**Stigma and power dynamics.** Stigma was noted as a possible limitation for co-design and co-creation research involving older adults living with HIV, and researchers used a variety of methods to overcome this potential barrier. For example, one study held separate workshops for those living with HIV and without, in view of ongoing stigma around HIV status in Tanzania [31]. Similarly, one study conducted in Europe pointed to challenges preventing participation in workshops for historically marginalized and stigmatized patients, resulting in individual interviews being offered as an alternative to workshops [35].

Four studies mentioned potential power imbalances between academic researchers and participants. In one study, the team limited the number of researchers present at study activities and asked participants whom they wanted involved to reduce power differentials and support informal discussion [34]. However, the remaining three studies did not discuss potential power imbalances in detail nor report observing this during the co-design or co-creation process [31,33,36].

**Partnership-specific challenges.** Partnership-based studies described feasibility and governance-related issues. In one study, researchers described that a partnership model required significant time investment and, given competing priorities for co-design partners and project complexity, timelines were at times unexpectedly extended [37]. Partners also described feeling an emotional burden when engaging with evidence related to aging and disease [37]. Researchers recommended clear terms of reference and communication strategies to manage differing levels of expertise and power dynamics, including situations where participants knew members of the CAB [36,37]. Maintaining strong levels of engagement among co-design partners required compensation and recognition [36] and partner skill-building; authors also recommended formal evaluation of the partnership itself to assess the value of community involvement [37].

**Sample size and reporting limitations.** A limitation noted by three studies was small sample size, which prevented data saturation and limited the generalizability of the findings [32–34]. Some researchers noted that they were unable to collect any demographic information beyond age, sex, and time living with HIV, which prevented their reporting of other characteristics that could have affected participant responses [33].

## Discussion

Our scoping review is the first to synthesize existing literature on the use of co-design and co-creation in research involving older adults living with HIV. Of the seven articles meeting our inclusion criteria, only two studies described partnership-based co-design [36,37] while the remaining five studies described consultation-based co-creation with study participants [31–35]. Our findings suggest that both co-design and co-creation were effective in eliciting perspectives, priorities, concerns, and suggestions from older adults living with HIV, with the additional benefits of increasing research participation and engagement from this patient population. However, the small body of evidence suggests a need for further research in this area or an expansion of the inclusion criteria. We noted several challenges and limitations across all studies, including those that were specific to partnership-based co-design. A strength of this review was its focus on older adults living with HIV, a population group with unique considerations for participatory research; the synthesis provided in this review contributes to the literature by identifying limitations in current research practices and by offering guidance for future co-design and co-creation research supporting older adults living with HIV.

### Recruitment and equity

Recruitment commonly relied on HIV clinics, community-based organizations, or researcher networks. Insights from our patient advisory committee suggest that these recruitment methods may inadvertently exclude more isolated or underserved populations, such as those experiencing stigma, housing instability, or limited access to healthcare. We further noted that, unless purposively sampled, studies would exclude participants of colour, people of lower socioeconomic status, rural residents, and those from lower- and middle-income countries. Advisory committee members emphasized the importance of diversifying recruitment strategies to non-traditional settings (e.g., community centers, faith-based organizations, digital platforms) and leveraging peer navigators or community health workers with lived experience to build trust and bridge engagement gaps. Local community partners were valuable for several stages of research including participant recruitment efforts (e.g., social media, posters) and facilitation support (e.g., translation), due to existing trust, confidentiality, and rapport with participants [35]. These insights underscore the need for more inclusive and tailored recruitment approaches to ensure that co-design and co-creation initiatives capture a broader range of experiences and perspectives from older adults living with HIV, ultimately enhancing the relevance and impact of interventions. Compared to consultation-only studies [31–35], the two partnership studies embedded community decision-making, but required more resources and role clarity [36,37], highlighting a unique trade-off in co-design approaches.

### Generalizability and transferability

Across studies, participants tended to be well-informed and already engaged in HIV education, services, or research, which limits applicability to those who have less access to care, are less familiar with research, are unable to engage in workshops or interviews, or are of lower health status. Likewise, the co-designed digital interventions may not be as widely applicable as predicted, due to the exclusion of individuals less familiar with technology during the co-design process. Our concerns over generalizability were further compounded by small sample sizes and geographic limitations in the studies largely to urban centres. While consultation-only designs may be easier to replicate across settings, partnership designs can strengthen feasibility and implementation planning if partners are given explicit decision-making roles and reflect those most affected [36,37].

### Gender and cultural context

Where reported, men were overrepresented, except in studies that purposively sampled women or were conducted in contexts where HIV prevalence was higher among women (e.g., Tanzania). Our advisory committee noted that gender dynamics and cultural contexts may influence participation and engagement rates. Consistent with previous studies, partnering with church-based networks may provide safer spaces for older Black women living with HIV given the role of religion and spirituality in resilience and engagement [44]. Future co-creation studies may thus benefit from tailoring study activities to suit the cultural preferences of their patient population, document how context shapes co-design and co-creation, and use culturally sensitive recruitment. Further, in the two studies that engaged partners an advisory capacity, few demographic details were provided. This limits a comprehensive understanding of who held decision-making power, whether equity-denied communities were represented among partners, and implementation feasibility. Future co-design work should prioritize collecting and reporting on partner-level characteristics using a PROGRESS-Plus lens [43], as well as details on selection criteria, roles, compensation, training, and retention to avoid tokenism.

### Duration and depth of involvement

Previous literature suggests that successful co-design and co-creation processes involve long-standing meaningful participant engagement and high participant satisfaction [45,46]. However, most included studies used one-time activities, which likely minimized retention or engagement challenges but limited depth of the data collected and failed to capture evolution of participants’ views over time. Most consultation-only studies relied on single-session activities, whereas one partnership study embedded older adults as ongoing research team partners across multiple stages [37]. To date, co-design research involving older adults living with HIV has focused more on research planning and less on evaluation and impact [31]. In practice, ongoing partnership can strengthen decisions, implementation planning, and continuity. Single-meeting CABs, as seen in one included study [36], are more feasible, but offer less decision-making influence for partners. There is thus the risk of low utility and sustainability for the co-designed interventions, in addition to lost potential benefits of sustaining long-term community-based partnerships. Future studies could re-engage co-design participants in analysis, implementation, evaluation, and dissemination, rather than limiting participation to the initial stages of conception and project planning. This is consistent with participant priorities for research education [31] and researcher recommendations for training opportunities and evaluation of the partnership process [37].

### Stigma, power, and partner well-being

Finally, our advisory committee emphasized that the emotional and psychological impact of stigma must be addressed in the co-design and co-creation process. During these activities, participants may experience heightened levels of vulnerability and distress when discussing their experiences with HIV, especially in public or group settings. In one included study, engaging with evidence on ageing and disease were described as partners by emotionally straining [37], highlighting the importance of proactive supports and clear boundaries during partnership activities. Our advisory committee suggested integrating mental health support into the co-design process, ensuring that participants have access to counseling or peer support groups before, during, and after participation. This support would help to alleviate the anxiety that may arise from discussing sensitive issues related to their health, thereby fostering a safer and more open environment for engagement. In partnership settings, these supports are particularly important due to longer timeframes, decision responsibilities, and potential power dynamics, while consultation-only studies may require briefer, but still proactive, supports.

### Implications for future co-design vs. consultation

The limitations and challenges we have identified offer insight into how future research could expand upon the current literature. Studies may benefit from more inclusive recruitment approaches, particularly through the involvement of local community partners, to reach historically and systemically marginalized groups and subsequently strengthen the generalizability of results and the quality of interventions. Participation options should be diversified (e.g., digital or anonymous avenues) to reduce barriers related to stigma, geography, time, digital literacy, or physical challenges such as reduced mobility and sensory impairment. As noted in one study, including healthcare providers and community agencies within co-design activities can support implementation planning [34]. Funding models should support long-term participant engagement in co-design and co-creation research, as the current predominance of short-term, single-session involvement undermines participatory aims by limiting end-user roles with little opportunity for involvement at later stages. While potential power imbalances were not widely reported by included studies, some researchers suggested that this could be overcome by giving study oversight to participants and by involving them as members of the research team in a model resembling community advisory boards, offering another potential strategy for future studies. Future co-design and co-creation research supporting older adults living with HIV should strive for meaningful involvement of participants early on, with shared decision-making, diverse representation, and careful consideration of potential barriers to participation.

### Study limitations

Our scoping review has several limitations. Our search criteria excluded articles that were not available in English, hence we may have excluded relevant literature presented in other languages and involving non-English-speaking older adults living with HIV. Moreover, the use of “co-design” and “co-creation” terminology is inconsistently used in the literature, although other similar terms related to participatory research may have been used by researchers instead. Consequently, our database and grey literature searches may have missed relevant research conducted with participatory methodology, but which was reported under other terminology. Furthermore, our inclusion and exclusion criteria may have been over-stringent, resulting in the low number of articles appropriate for inclusion. Lastly, although the focus of our scoping review was on study participants who identified as older adults living with HIV aged 50 or older, studies included participants of a wider age range, and we were unable to stratify participant characteristics or study findings based on age, thus our findings may not completely align with our targeted participant age range.

## Conclusions

In conclusion, this scoping review synthesized existing literature on the use of co-design and co-creation approaches involving older adults living with HIV. Engagement was skewed to co-creation approaches, predominantly utilizing workshops, focus groups, and interviews, while partnership was only described in two of the seven included studies. Both were valuable for developing interventions and research agendas that address the chronic health concerns of ageing populations living with HIV. However, the short-term nature of consultation-based engagement and recruitment strategies that favored well-informed individuals limit the generalizability of the findings to the broader older adults living with HIV community. Despite these limitations, both co-creation and co-design facilitated meaningful participation, drawing on the lived experiences of older adults living with HIV to inform relevant and context-specific solutions. The review also highlighted the importance of involving community partners to enhance recruitment and participant engagement.

Future research should explore more inclusive recruitment strategies, for partners and participants alike, to reach historically excluded groups within the older adults living with HIV population and consider longer-term designs to deepen participant involvement and sustain intervention impact. Additionally, employing digital participation could enhance accessibility and engagement. This review underscores the potential of co-design and co-creation in addressing the evolving needs of older adults living with HIV and advocates for continued methodological innovation and inclusivity to better support this ageing population. By advancing co-design and co-creation practices, future studies can contribute to more equitable and effective health interventions for older adults living with HIV.

## Supporting information

S1 Checklist PRISMA-ScR checklist. (DOCX)

S1 Appendix Full database search strategies and dates. (DOCX)
