# Supplementary material for: The role of co-design and co-creation methodologies in research supporting older adults living with HIV: A scoping review
Source: PLoS One. 2026 Sep 18;21(9):e0358263. doi: 10.1371/journal.pone.0358263 (PMC13588517; doi:10.1371/journal.pone.0358263)
Supplement: Supplementary file 2 [file pone.0358263.s002.docx]

# All search strategies run and exported on May 17, 2024.

| **Databases** | **Number of Results** |
| --- | --- |
| OVID Medline ® All | 571 |
| OVID Embase+Embase Classic | 1526 |
| EBSCO CINAHL Complete | 214 |
| NLM PubMed (Not including Medline) | 12 |
| Clarivate Web of Science including:  - WOS.SCI: 1900 to 2024  - WOS.AHCI: 1975 to 2024  - WOS.BHCI: 2005 to 2024  - WOS.BSCI: 2005 to 2024  - WOS.ESCI: 2005 to 2024  - WOS.ISTP: 1990 to 2024  - WOS.SSCI: 1900 to 2024  - WOS.ISSHP: 1990 to 2024 | 158 |
| Scopus | 101 |
| **Total Results Before Deduplication** | 2582 |
| **Total Results After Deduplication in Covidence**  **(Uploaded to Covidence on May 17, 2024)** | 1973  609 duplicates removed |

## OVID Medline ® All:

Ovid MEDLINE(R) ALL <1946 to May 16, 2024>

1	hiv/ or hiv-1/ or hiv-2/	109160

2	hiv infections/ or acquired immunodeficiency syndrome/ or acute retroviral syndrome/ or aids arteritis, central nervous system/ or aids dementia complex/ or aids-associated nephropathy/ or aids-related complex/ or aids-related opportunistic infections/ or hiv enteropathy/ or hiv seropositivity/ or hiv wasting syndrome/ or hiv-associated lipodystrophy syndrome/	322587

3	(HIV or (human adj1 immunodeficien*) or (human adj1 immune adj1 deficien*)).tw,kf,kw.	386631

4	((AIDS adj1 virus) or (acquired immune adj1 deficien*) or (acquired adj1 immunodeficien*)).tw,kf,kw.	29244

5	1 or 2 or 3 or 4	463696

6	aged/ or "aged, 80 and over"/ or centenarians/ or nonagenarians/ or octogenarians/ or middle aged/	5632889

7	((Old adj2 Adult*) or (Old adj2 Person*) or (Old adj2 People*) or (Old adj2 Patient*) or (Old adj2 Citizen*) or (Older adj2 Adult*) or (Older adj2 Person*) or (Older adj2 People*) or (Older adj2 Patient*) or (Older adj2 Citizen*) or (Oldest adj2 Adult*) or (Oldest adj2 Person*) or (Oldest adj2 People*) or (Oldest adj2 Patient*) or (Oldest adj2 Citizen*) or (Ag?ing adj2 Adult*) or (Ag?ing adj2 Person*) or (Ag?ing adj2 People*) or (Ag?ing adj2 Patient*) or (Ag?ing adj2 Citizen*) or (Aged adj2 Adult*) or (Aged adj2 Person*) or (Aged adj2 People*) or (Aged adj2 Patient*) or (Aged adj2 Citizen*)).tw,kf,kw.	527877

8	(Oldest Old or Elder* or Geriatric* or Senior* or Long-Lived or Over-the-hill or Senescen* or Centenarian or Nonagenarian or Octogenarian or Septuagenarian or Sexagenerian or Unyoung).tw,kf,kw.	508667

9	((Aged adj "50") or (Aged adj "60") or (Aged adj "70") or (Aged adj "75") or (Aged adj "80") or (Aged adj "85") or (Aged adj "90") or (Aged adj "95") or (Aged adj "100") or (Aged adj fifty) or (Aged adj sixty) or (Aged adj fifty-five) or (Aged adj sixty-five) or (Aged adj seventy) or (Aged adj seventy-five) or (Aged adj eighty) or (Aged adj eighty-five) or (Aged adj ninety) or (Aged adj ninety-five) or (Aged adj one hundred) or (Aged adj a hundred)).tw,kf,kw.	70538

10	6 or 7 or 8 or 9	6040041

11	Research Design/	128036

12	Community-Based Participatory Research/	5836

13	(Co-design* or "Co design" or Codesign* or Co-creation* or "Co creation" or Cocreation* or Co-production* or "Co production" or Coproduction*).tw,kf,kw.	9739

14	((communitybased adj2 research*) or (community-based adj2 research*) or (community-based adj2 education*) or (community-based adj1 program*) or (consumer driven adj2 research*) or (community adj1 engage*) or (community-engaged adj1 research*) or (consumer-driven adj2 research)).tw,kf,kw.	16444

15	((participatory adj1 research?) or (participatory adj1 arts adj1 engage*) or (community adj1 engage* adj1 research*)).tw,kf,kw.	7222

16	11 or 12 or 13 or 14 or 15	157195

17	5 and 10 and 16	571

## OVID Embase+Embase Classic:

Embase Classic+Embase <1947 to 2024 May 16>

1	human immunodeficiency virus/ or human immunodeficiency virus 1/ or human immunodeficiency virus 2/ or acquired immune deficiency syndrome/ or acute HIV infection/ or AIDS arteritis/ or HIV associated dementia/ or HIV associated nephropathy/ or AIDS related complex/ or AIDS-related opportunistic infections/ or HIV enteropathy/ or Human immunodeficiency virus infection/ or HIV wasting syndrome/ or HIV associated lipodystrophy/	543266

2	(HIV or (human adj1 immunodeficien*) or (human adj1 immune adj1 deficien*)).tw,kf,kw.	499841

3	((AIDS adj1 virus) or (acquired immune adj1 deficien*) or (acquired adj1 immunodeficien*)).tw,kf,kw.	30131

4	1 or 2 or 3	639977

5	aged/	4010540

6	very elderly/	315786

7	middle aged/	2371709

8	((Old adj2 Adult*) or (Old adj2 Person*) or (Old adj2 People*) or (Old adj2 Patient*) or (Old adj2 Citizen*) or (Older adj2 Adult*) or (Older adj2 Person*) or (Older adj2 People*) or (Older adj2 Patient*) or (Older adj2 Citizen*) or (Oldest adj2 Adult*) or (Oldest adj2 Person*) or (Oldest adj2 People*) or (Oldest adj2 Patient*) or (Oldest adj2 Citizen*) or (Ag?ing adj2 Adult*) or (Ag?ing adj2 Person*) or (Ag?ing adj2 People*) or (Ag?ing adj2 Patient*) or (Ag?ing adj2 Citizen*) or (Aged adj2 Adult*) or (Aged adj2 Person*) or (Aged adj2 People*) or (Aged adj2 Patient*) or (Aged adj2 Citizen*)).tw,kf,kw.	768048

9	(Oldest Old or Elder* or Geriatric* or Senior* or Long-Lived or Over-the-hill or Senescen* or Centenarian or Nonagenarian or Octogenarian or Septuagenarian or Sexagenerian or Unyoung).tw,kf,kw.	712349

10	((Aged adj "50") or (Aged adj "60") or (Aged adj "70") or (Aged adj "75") or (Aged adj "80") or (Aged adj "85") or (Aged adj "90") or (Aged adj "95") or (Aged adj "100") or (Aged adj fifty) or (Aged adj sixty) or (Aged adj fifty-five) or (Aged adj sixty-five) or (Aged adj seventy) or (Aged adj seventy-five) or (Aged adj eighty) or (Aged adj eighty-five) or (Aged adj ninety) or (Aged adj ninety-five) or (Aged adj one hundred) or (Aged adj a hundred)).tw,kf,kw.	101324

11	6 or 7 or 8 or 9 or 10	3691204

12	methodology/	1690647

13	participatory research/	7704

14	(Co-design* or "Co design" or Codesign* or Co-creation* or "Co creation" or Cocreation* or Co-production* or "Co production" or Coproduction*).tw,kf,kw.	11355

15	((communitybased adj2 research*) or (community-based adj2 research*) or (community-based adj2 education*) or (community-based adj1 program*) or (consumer driven adj2 research*) or (community adj1 engage*) or (community-engaged adj1 research*) or (consumer-driven adj2 research)).tw,kf,kw.	19390

16	((participatory adj1 research?) or (participatory adj1 arts adj1 engage*) or (community adj1 engage* adj1 research*)).tw,kf,kw.	7996

17	12 or 13 or 14 or 15 or 16	1723626

18	4 and 11 and 17	1526

## NLM’s PubMed (Not including Medline):

((((((hiv[MeSH Terms]) or (hiv-1[MeSH Terms]) or (hiv-2[MeSH Terms])) OR ((hiv infections[MeSH Terms]) or (acquired immunodeficiency syndrome[MeSH Terms]) or (acute retroviral syndrome[MeSH Terms]) or (aids arteritis, central nervous system[MeSH Terms]) or (aids dementia complex[MeSH Terms]) or (aids-associated nephropathy[MeSH Terms]) or (aids-related complex[MeSH Terms]) or (aids-related opportunistic infections[MeSH Terms]) or (hiv enteropathy[MeSH Terms]) or (hiv seropositivity[MeSH Terms]) or (hiv wasting syndrome[MeSH Terms]) or (hiv-associated lipodystrophy syndrome[MeSH Terms]))) OR (HIV[Title/Abstract])) OR (("AIDS virus"[Title/Abstract:~1]) OR ("acquired immune deficiency"[Title/Abstract:~1]) OR ("acquired immunodeficiency"[Title/Abstract:~1]) OR ("acquired immunodeficiencies"[Title/Abstract:~1]))) AND (((((aged[MeSH Terms]) or ("aged, 80 and over"[MeSH Terms]) or (centenarians[MeSH Terms]) or (nonagenarians[MeSH Terms]) or (octogenarians[MeSH Terms]) or (middle aged[MeSH Terms])) OR ((Old Adult*[Title/Abstract]) OR (Old Person*[Title/Abstract]) OR (Old People*[Title/Abstract]) OR (Old Patient*[Title/Abstract]) OR (Old Citizen*[Title/Abstract]) OR (Older Adult*[Title/Abstract]) OR (Older Person*[Title/Abstract]) OR (Older People*[Title/Abstract]) OR (Older Patient*[Title/Abstract]) OR (Older Citizen*[Title/Abstract]) OR (Oldest Adult*[Title/Abstract]) OR (Oldest Person*[Title/Abstract]) OR (Oldest People*[Title/Abstract]) OR (Oldest Patient*[Title/Abstract]) OR (Oldest Citizen*[Title/Abstract]) OR (Aging Adult*[Title/Abstract]) OR (Aging Person*[Title/Abstract]) OR (Aging People*[Title/Abstract]) OR (Aging Patient*[Title/Abstract]) OR (Aging Citizen*[Title/Abstract]) OR (Aged Adult*[Title/Abstract]) OR (Aged Person*[Title/Abstract]) OR (Aged People*[Title/Abstract]) OR (Aged Patient*[Title/Abstract]) OR (Aged Citizen*[Title/Abstract]))) OR (Oldest Old[Title/Abstract] OR Elder*[Title/Abstract] OR Geriatric*[Title/Abstract] OR Senior*[Title/Abstract] OR Long-Lived[Title/Abstract] OR Over-the-hill[Title/Abstract] OR Senescen*[Title/Abstract] OR Centenarian[Title/Abstract] OR Nonagenarian[Title/Abstract] OR Octogenarian[Title/Abstract] OR Septuagenarian[Title/Abstract] OR Sexagenerian[Title/Abstract] OR Unyoung[Title/Abstract])) OR ((Aged "50"[Title/Abstract]) OR (Aged "60"[Title/Abstract]) OR (Aged "70"[Title/Abstract]) OR (Aged "75"[Title/Abstract]) OR (Aged "80"[Title/Abstract]) OR (Aged "85"[Title/Abstract]) OR (Aged "90"[Title/Abstract]) OR (Aged "95"[Title/Abstract]) OR (Aged "100"[Title/Abstract]) OR (Aged fifty[Title/Abstract]) OR (Aged sixty[Title/Abstract]) OR (Aged fifty-five[Title/Abstract]) OR (Aged sixty-five[Title/Abstract]) OR (Aged seventy[Title/Abstract]) OR (Aged seventy-five[Title/Abstract]) OR (Aged eighty[Title/Abstract]) OR (Aged eighty-five[Title/Abstract]) OR (Aged ninety[Title/Abstract]) OR (Aged ninety-five[Title/Abstract]) OR (Aged one hundred[Title/Abstract]) OR (Aged a hundred[Title/Abstract])))) AND (((((Research Design[MeSH Terms]) OR (Community-Based Participatory Research[MeSH Terms])) OR (Co-design*[Title/Abstract] OR "Co design"[Title/Abstract] OR Codesign*[Title/Abstract] OR Co-creation*[Title/Abstract] OR "Co creation"[Title/Abstract] OR Cocreation*[Title/Abstract] OR Co-production*[Title/Abstract] OR "Co production"[Title/Abstract] OR Coproduction[Title/Abstract])) OR ((communitybased research*[Title/Abstract]) OR (community-based research*[Title/Abstract]) OR (community-based education*[Title/Abstract]) OR (community-based program*[Title/Abstract]) OR (consumer driven research*[Title/Abstract]) OR (community engage*[Title/Abstract]) OR (community-engaged research*[Title/Abstract]) OR (consumer-driven research[Title/Abstract]))) OR ((participatory research[Title/Abstract]) OR (participatory arts engage*[Title/Abstract]) OR (community engage* research*[Title/Abstract]))) AND (pubstatusaheadofprint OR publisher[SB] OR in process[SB] OR pubmednotmedline[SB])

**Results: 12**

## EBSCOhost’s CINAHL:

Fri, May 17, 2024 1:30:52 PM

| **#** | **Query** | **Limiters/Expanders** | **Last Run Via** | **Results** |
| --- | --- | --- | --- | --- |
| S16 | S5 AND S10 AND S15 | Expanders - Apply equivalent subjects  Search modes - Boolean/Phrase | Interface - EBSCOhost Research Databases  Search Screen - Advanced Search  Database - CINAHL Complete | 214 |
| S15 | S11 OR S12 OR S13 OR S14 | Expanders - Apply equivalent subjects  Search modes - Boolean/Phrase | Interface - EBSCOhost Research Databases  Search Screen - Advanced Search  Database - CINAHL Complete | 50,425 |
| S14 | TI ( (participatory N0 research#) or (participatory N0 arts N0 engage*) or (community N0 engage* N0 research*) ) OR AB ( (participatory N0 research#) or (participatory N0 arts N0 engage*) or (community N0 engage* N0 research*) ) | Expanders - Apply equivalent subjects  Search modes - Boolean/Phrase | Interface - EBSCOhost Research Databases  Search Screen - Advanced Search  Database - CINAHL Complete | 3,529 |
| S13 | TI ( (communitybased N1 research*) or (community-based N1 research*) or (community-based N1 education*) or (community-based N0 program*) or (consumer driven N1 research*) or (community N0 engage*) or (community-engaged N0 research*) or (consumer-driven N1 research) ) OR AB ( (communitybased N1 research*) or (community-based N1 research*) or (community-based N1 education*) or (community-based N0 program*) or (consumer driven N1 research*) or (community N0 engage*) or (community-engaged N0 research*) or (consumer-driven N1 research) ) | Expanders - Apply equivalent subjects  Search modes - Boolean/Phrase | Interface - EBSCOhost Research Databases  Search Screen - Advanced Search  Database - CINAHL Complete | 8,174 |
| S12 | TI ( (Co-design* or "Co design" or Codesign* or Co-creation* or "Co creation" or Cocreation* or Co-production* or "Co production" or Coproduction*) ) OR AB ( (Co-design* or "Co design" or Codesign* or Co-creation* or "Co creation" or Cocreation* or Co-production* or "Co production" or Coproduction*) ) | Expanders - Apply equivalent subjects  Search modes - Boolean/Phrase | Interface - EBSCOhost Research Databases  Search Screen - Advanced Search  Database - CINAHL Complete | 3,945 |
| S11 | (MH "Study Design") | Expanders - Apply equivalent subjects  Search modes - Boolean/Phrase | Interface - EBSCOhost Research Databases  Search Screen - Advanced Search  Database - CINAHL Complete | 37,610 |
| S10 | S6 OR S7 OR S8 OR S9 | Expanders - Apply equivalent subjects  Search modes - Boolean/Phrase | Interface - EBSCOhost Research Databases  Search Screen - Advanced Search  Database - CINAHL Complete | 1,587,286 |
| S9 | TI ( (Aged N0 "50") or (Aged N0 "60") or (Aged N0 "70") or (Aged N0 "75") or (Aged N0 "80") or (Aged N0 "85") or (Aged N0 "90") or (Aged N0 "95") or (Aged N0 "100") or (Aged N0 fifty) or (Aged N0 sixty) or (Aged N0 fifty-five) or (Aged N0 sixty-five) or (Aged N0 seventy) or (Aged N0 seventy-five) or (Aged N0 eighty) or (Aged N0 eighty-five) or (Aged N0 ninety) or (Aged N0 ninety-five) or (Aged N0 one hundred) or (Aged N0 a hundred) ) OR AB ( (Aged N0 "50") or (Aged N0 "60") or (Aged N0 "70") or (Aged N0 "75") or (Aged N0 "80") or (Aged N0 "85") or (Aged N0 "90") or (Aged N0 "95") or (Aged N0 "100") or (Aged N0 fifty) or (Aged N0 sixty) or (Aged N0 fifty-five) or (Aged N0 sixty-five) or (Aged N0 seventy) or (Aged N0 seventy-five) or (Aged N0 eighty) or (Aged N0 eighty-five) or (Aged N0 ninety) or (Aged N0 ninety-five) or (Aged N0 one hundred) or (Aged N0 a hundred) ) | Expanders - Apply equivalent subjects  Search modes - Boolean/Phrase | Interface - EBSCOhost Research Databases  Search Screen - Advanced Search  Database - CINAHL Complete | 22,754 |
| S8 | TI ( Oldest Old or Elder* or Geriatric* or Senior* or Long-Lived or Over-the-hill or Senescen* or Centenarian or Nonagenarian or Octogenarian or Septuagenarian or Sexagenerian or Unyoung ) OR AB ( Oldest Old or Elder* or Geriatric* or Senior* or Long-Lived or Over-the-hill or Senescen* or Centenarian or Nonagenarian or Octogenarian or Septuagenarian or Sexagenerian or Unyoung ) | Expanders - Apply equivalent subjects  Search modes - Boolean/Phrase | Interface - EBSCOhost Research Databases  Search Screen - Advanced Search  Database - CINAHL Complete | 171,826 |
| S7 | TI ( (Old N1 Adult*) or (Old N1 Person*) or (Old N1 People*) or (Old N1 Patient*) or (Old N1 Citizen*) or (Older N1 Adult*) or (Older N1 Person*) or (Older N1 People*) or (Older N1 Patient*) or (Older N1 Citizen*) or (Oldest N1 Adult*) or (Oldest N1 Person*) or (Oldest N1 People*) or (Oldest N1 Patient*) or (Oldest N1 Citizen*) or (Ag#ing N1 Adult*) or (Ag#ing N1 Person*) or (Ag#ing N1 People*) or (Ag#ing N1 Patient*) or (Ag#ing N1 Citizen*) or (Aged N1 Adult*) or (Aged N1 Person*) or (Aged N1 People*) or (Aged N1 Patient*) or (Aged N1 Citizen*) ) OR AB ( (Old N1 Adult*) or (Old N1 Person*) or (Old N1 People*) or (Old N1 Patient*) or (Old N1 Citizen*) or (Older N1 Adult*) or (Older N1 Person*) or (Older N1 People*) or (Older N1 Patient*) or (Older N1 Citizen*) or (Oldest N1 Adult*) or (Oldest N1 Person*) or (Oldest N1 People*) or (Oldest N1 Patient*) or (Oldest N1 Citizen*) or (Ag#ing N1 Adult*) or (Ag#ing N1 Person*) or (Ag#ing N1 People*) or (Ag#ing N1 Patient*) or (Ag#ing N1 Citizen*) or (Aged N1 Adult*) or (Aged N1 Person*) or (Aged N1 People*) or (Aged N1 Patient*) or (Aged N1 Citizen*) ) | Expanders - Apply equivalent subjects  Search modes - Boolean/Phrase | Interface - EBSCOhost Research Databases  Search Screen - Advanced Search  Database - CINAHL Complete | 196,882 |
| S6 | (MH "Aged") OR (MH "Aged, 80 and Over") OR (MH "Centenarians") OR (MH "Nonagenarians") OR (MH "Octogenarians") OR (MH "Middle Age") | Expanders - Apply equivalent subjects  Search modes - Boolean/Phrase | Interface - EBSCOhost Research Databases  Search Screen - Advanced Search  Database - CINAHL Complete | 1,474,929 |
| S5 | S1 OR S2 OR S3 OR S4 | Expanders - Apply equivalent subjects  Search modes - Boolean/Phrase | Interface - EBSCOhost Research Databases  Search Screen - Advanced Search  Database - CINAHL Complete | 136,209 |
| S4 | TI ( (AIDS N0 virus) or (acquired immune N0 deficien*) or (acquired N0 immunodeficien*) ) OR AB ( (AIDS N0 virus) or (acquired immune N0 deficien*) or (acquired N0 immunodeficien*) ) | Expanders - Apply equivalent subjects  Search modes - Boolean/Phrase | Interface - EBSCOhost Research Databases  Search Screen - Advanced Search  Database - CINAHL Complete | 3,974 |
| S3 | TI ( HIV or (human N0 immunodeficien*) or (human N0 immune N0 deficien*) ) OR AB ( HIV or (human N0 immunodeficien*) or (human N0 immune N0 deficien*) ) | Expanders - Apply equivalent subjects  Search modes - Boolean/Phrase | Interface - EBSCOhost Research Databases  Search Screen - Advanced Search  Database - CINAHL Complete | 109,808 |
| S2 | (MH "HIV Infections") OR (MH "Acquired Immunodeficiency Syndrome") OR (MH "AIDS Dementia Complex") OR (MH "AIDS-Associated Nephropathy") OR (MH "AIDS-Related Complex") OR (MH "AIDS-Related Opportunistic Infections") OR (MH "HIV Enteropathy") OR (MH "HIV Seropositivity") OR (MH "HIV Wasting Syndrome") | Expanders - Apply equivalent subjects  Search modes - Boolean/Phrase | Interface - EBSCOhost Research Databases  Search Screen - Advanced Search  Database - CINAHL Complete | 95,996 |
| S1 | (MH "Human Immunodeficiency Virus") OR (MH "HIV-1") | Expanders - Apply equivalent subjects  Search modes - Boolean/Phrase | Interface - EBSCOhost Research Databases  Search Screen - Advanced Search  Database - CINAHL Complete | 11,254 |

## Clarivate’s Web of Science:

| Entitlements | # | Search Query | Database | Results | Date Run |
| --- | --- | --- | --- | --- | --- |
| - WOS.SCI: 1900 to 2024  - WOS.AHCI: 1975 to 2024  - WOS.BHCI: 2005 to 2024  - WOS.BSCI: 2005 to 2024  - WOS.ESCI: 2005 to 2024  - WOS.ISTP: 1990 to 2024  - WOS.SSCI: 1900 to 2024  - WOS.ISSHP: 1990 to 2024 | 1 | HIV or (human Near/0 immunodeficien*) or (human Near/0 immune Near/0 deficien*) (Topic) | Web of Science Core Collection | 479838 | Fri May 17 2024 13:44:35 |
| - WOS.SCI: 1900 to 2024  - WOS.AHCI: 1975 to 2024  - WOS.BHCI: 2005 to 2024  - WOS.BSCI: 2005 to 2024  - WOS.ESCI: 2005 to 2024  - WOS.ISTP: 1990 to 2024  - WOS.SSCI: 1900 to 2024  - WOS.ISSHP: 1990 to 2024 | 2 | TS=((AIDS Near/0 virus) or (acquired immune Near/0 deficien*) or (acquired Near/0 immunodeficien*)) | Web of Science Core Collection | 34818 | Fri May 17 2024 13:49:39 |
| - WOS.SCI: 1900 to 2024  - WOS.AHCI: 1975 to 2024  - WOS.BHCI: 2005 to 2024  - WOS.BSCI: 2005 to 2024  - WOS.ESCI: 2005 to 2024  - WOS.ISTP: 1990 to 2024  - WOS.SSCI: 1900 to 2024  - WOS.ISSHP: 1990 to 2024 | 3 | #2 OR #1 | Web of Science Core Collection | 494402 | Fri May 17 2024 13:49:44 |
| - WOS.SCI: 1900 to 2024  - WOS.AHCI: 1975 to 2024  - WOS.BHCI: 2005 to 2024  - WOS.BSCI: 2005 to 2024  - WOS.ESCI: 2005 to 2024  - WOS.ISTP: 1990 to 2024  - WOS.SSCI: 1900 to 2024  - WOS.ISSHP: 1990 to 2024 | 4 | TS=((Old Near/1 Adult*) or (Old Near/1 Person*) or (Old Near/1 People*) or (Old Near/1 Patient*) or (Old Near/1 Citizen*) or (Older Near/1 Adult*) or (Older Near/1 Person*) or (Older Near/1 People*) or (Older Near/1 Patient*) or (Older Near/1 Citizen*) or (Oldest Near/1 Adult*) or (Oldest Near/1 Person*) or (Oldest Near/1 People*) or (Oldest Near/1 Patient*) or (Oldest Near/1 Citizen*) or (Ag$ing Near/1 Adult*) or (Ag$ing Near/1 Person*) or (Ag$ing Near/1 People*) or (Ag$ing Near/1 Patient*) or (Ag$ing Near/1 Citizen*) or (Aged Near/1 Adult*) or (Aged Near/1 Person*) or (Aged Near/1 People*) or (Aged Near/1 Patient*) or (Aged Near/1 Citizen*)) | Web of Science Core Collection | 822190 | Fri May 17 2024 13:54:18 |
| - WOS.SCI: 1900 to 2024  - WOS.AHCI: 1975 to 2024  - WOS.BHCI: 2005 to 2024  - WOS.BSCI: 2005 to 2024  - WOS.ESCI: 2005 to 2024  - WOS.ISTP: 1990 to 2024  - WOS.SSCI: 1900 to 2024  - WOS.ISSHP: 1990 to 2024 | 5 | TS=(Oldest Old or Elder* or Geriatric* or Senior* or Long-Lived or Over-the-hill or Senescen* or Centenarian or Nonagenarian or Octogenarian or Septuagenarian or Sexagenerian or Unyoung) | Web of Science Core Collection | 2797422 | Fri May 17 2024 13:54:28 |
| - WOS.SCI: 1900 to 2024  - WOS.AHCI: 1975 to 2024  - WOS.BHCI: 2005 to 2024  - WOS.BSCI: 2005 to 2024  - WOS.ESCI: 2005 to 2024  - WOS.ISTP: 1990 to 2024  - WOS.SSCI: 1900 to 2024  - WOS.ISSHP: 1990 to 2024 | 6 | TS=((Aged Near/0 "50") or (Aged Near/0 "60") or (Aged Near/0 "70") or (Aged Near/0 "75") or (Aged Near/0 "80") or (Aged Near/0 "85") or (Aged Near/0 "90") or (Aged Near/0 "95") or (Aged Near/0 "100") or (Aged Near/0 fifty) or (Aged Near/0 sixty) or (Aged Near/0 fifty-five) or (Aged Near/0 sixty-five) or (Aged Near/0 seventy) or (Aged Near/0 seventy-five) or (Aged Near/0 eighty) or (Aged Near/0 eighty-five) or (Aged Near/0 ninety) or (Aged Near/0 ninety-five) or (Aged Near/0 one hundred) or (Aged Near/0 a hundred)) | Web of Science Core Collection | 141257 | Fri May 17 2024 13:54:39 |
| - WOS.SCI: 1900 to 2024  - WOS.AHCI: 1975 to 2024  - WOS.BHCI: 2005 to 2024  - WOS.BSCI: 2005 to 2024  - WOS.ESCI: 2005 to 2024  - WOS.ISTP: 1990 to 2024  - WOS.SSCI: 1900 to 2024  - WOS.ISSHP: 1990 to 2024 | 7 | #6 OR #5 OR #4 | Web of Science Core Collection | 3126157 | Fri May 17 2024 13:54:53 |
| - WOS.SCI: 1900 to 2024  - WOS.AHCI: 1975 to 2024  - WOS.BHCI: 2005 to 2024  - WOS.BSCI: 2005 to 2024  - WOS.ESCI: 2005 to 2024  - WOS.ISTP: 1990 to 2024  - WOS.SSCI: 1900 to 2024  - WOS.ISSHP: 1990 to 2024 | 8 | TS=(Co-design* or "Co design" or Codesign* or Co-creation* or "Co creation" or Cocreation* or Co-production* or "Co production" or Coproduction*) | Web of Science Core Collection | 49296 | Fri May 17 2024 13:55:08 |
| - WOS.SCI: 1900 to 2024  - WOS.AHCI: 1975 to 2024  - WOS.BHCI: 2005 to 2024  - WOS.BSCI: 2005 to 2024  - WOS.ESCI: 2005 to 2024  - WOS.ISTP: 1990 to 2024  - WOS.SSCI: 1900 to 2024  - WOS.ISSHP: 1990 to 2024 | 9 | TS=((communitybased Near/1 research*) or (community-based Near/1 research*) or (community-based Near/1 education*) or (community-based Near/0 program*) or (consumer driven Near/1 research*) or (community Near/0 engage*) or (community-engaged Near/0 research*) or (consumer-driven Near/1 research)) | Web of Science Core Collection | 27730 | Fri May 17 2024 13:55:17 |
| - WOS.SCI: 1900 to 2024  - WOS.AHCI: 1975 to 2024  - WOS.BHCI: 2005 to 2024  - WOS.BSCI: 2005 to 2024  - WOS.ESCI: 2005 to 2024  - WOS.ISTP: 1990 to 2024  - WOS.SSCI: 1900 to 2024  - WOS.ISSHP: 1990 to 2024 | 10 | TS=((participatory Near/0 research$) or (participatory Near/0 arts Near/0 engage*) or (community Near/0 engage* Near/0 research*)) | Web of Science Core Collection | 14200 | Fri May 17 2024 13:55:27 |
| - WOS.SCI: 1900 to 2024  - WOS.AHCI: 1975 to 2024  - WOS.BHCI: 2005 to 2024  - WOS.BSCI: 2005 to 2024  - WOS.ESCI: 2005 to 2024  - WOS.ISTP: 1990 to 2024  - WOS.SSCI: 1900 to 2024  - WOS.ISSHP: 1990 to 2024 | 11 | #10 OR #9 OR #8 | Web of Science Core Collection | 82873 | Fri May 17 2024 13:55:35 |
| - WOS.SCI: 1900 to 2024  - WOS.AHCI: 1975 to 2024  - WOS.BHCI: 2005 to 2024  - WOS.BSCI: 2005 to 2024  - WOS.ESCI: 2005 to 2024  - WOS.ISTP: 1990 to 2024  - WOS.SSCI: 1900 to 2024  - WOS.ISSHP: 1990 to 2024 | 12 | #11 AND #7 AND #3 | Web of Science Core Collection | 158 | Fri May 17 2024 13:55:46 |

## Elsevier’s Scopus:

( ( TITLE-ABS-KEY ( ( participatory W/0 research* ) OR ( participatory W/0 arts W/0 engage* ) OR ( community W/0 engage* W/0 research* ) ) ) OR ( TITLE-ABS-KEY ( ( communitybased W/1 research* ) OR ( community-based W/1 research* ) OR ( community-based W/1 education* ) OR ( community-based W/0 program* ) OR ( consumer AND driven W/1 research* ) OR ( community W/0 engage* ) OR ( community-engaged W/0 research* ) OR ( consumer-driven W/1 research ) ) ) OR ( TITLE-ABS-KEY ( co-design* OR "Co design" OR codesign* OR co-creation* OR "Co creation" OR cocreation* OR co-production* OR "Co production" OR coproduction* ) ) ) AND ( ( TITLE-ABS-KEY ( ( aged W/0 "50" ) OR ( aged W/0 "60" ) OR ( aged W/0 "70" ) OR ( aged W/0 "75" ) OR ( aged W/0 "80" ) OR ( aged W/0 "85" ) OR ( aged W/0 "90" ) OR ( aged W/0 "95" ) OR ( aged W/0 "100" ) OR ( aged W/0 fifty ) OR ( aged W/0 sixty ) OR ( aged W/0 fifty-five ) OR ( aged W/0 sixty-five ) OR ( aged W/0 seventy ) OR ( aged W/0 seventy-five ) OR ( aged W/0 eighty ) OR ( aged W/0 eighty-five ) OR ( aged W/0 ninety ) OR ( aged W/0 ninety-five ) OR ( aged W/0 one AND hundred ) OR ( aged W/0 a AND hundred ) ) ) OR ( TITLE-ABS-KEY ( oldest AND old OR elder* OR geriatric* OR senior* OR long-lived OR over-the-hill OR senescen* OR centenarian OR nonagenarian OR octogenarian OR septuagenarian OR sexagenerian OR unyoung ) ) OR ( TITLE-ABS-KEY ( ( old W/1 adult* ) OR ( old W/1 person* ) OR ( old W/1 people* ) OR ( old W/1 patient* ) OR ( old W/1 citizen* ) OR ( older W/1 adult* ) OR ( older W/1 person* ) OR ( older W/1 people* ) OR ( older W/1 patient* ) OR ( older W/1 citizen* ) OR ( oldest W/1 adult* ) OR ( oldest W/1 person* ) OR ( oldest W/1 people* ) OR ( oldest W/1 patient* ) OR ( oldest W/1 citizen* ) OR ( ag*ing W/1 adult* ) OR ( ag*ing W/1 person* ) OR ( ag*ing W/1 people* ) OR ( ag*ing W/1 patient* ) OR ( ag*ing W/1 citizen* ) OR ( aged W/1 adult* ) OR ( aged W/1 person* ) OR ( aged W/1 people* ) OR ( aged W/1 patient* ) OR ( aged W/1 citizen* ) ) ) ) AND ( ( TITLE-ABS-KEY ( ( ( aids W/0 virus ) OR ( acquired AND immune W/0 deficien* ) OR ( acquired W/0 immunodeficien* ) ) ) ) OR ( TITLE-ABS-KEY ( ( hiv OR ( human W/0 immunodeficien* ) OR ( human W/0 immune W/0 deficien* ) ) ) ) )

**Results: 101**
